# A Novel Fluidic Platform for Semi-Automated Cell Culture into Multiwell-like Bioreactors

**DOI:** 10.3390/mi13070994

**Published:** 2022-06-24

**Authors:** Francesca Maria Orecchio, Vito Tommaso, Tommaso Santaniello, Sara Castiglioni, Federico Pezzotta, Andrea Monti, Francesco Butera, Jeanette Anne Marie Maier, Paolo Milani

**Affiliations:** 1 Interdisciplinary Centre of Excellence for Nanostructured Materials and Interfaces (C.I.Ma.I.Na.), Department of Physics, University of Milan, Via Giovanni Celoria, 16, 20133 Milan, Italy; francesca.orecchio@unimi.it (F.M.O.); federico.pezzotta@unimi.it (F.P.); jeanette.maier@unimi.it (J.A.M.M.); paolo.milani@mi.infn.it (P.M.); 2 Department of Biomedical and Clinical Sciences, University of Milan, Via Giovanni Battista Grassi 74, 20157 Milan, Italy; vito.tommaso@unimi.it (V.T.); sara.castiglioni@unimi.it (S.C.); 3 Dolphin Fluidics S.r.l., Via Leonardo Da Vinci, 40, 20094 Corsico, Italy; a.monti@dolphinfluidics.com (A.M.); f.butera@dolphinfluidics.com (F.B.)

**Keywords:** cells-on-a-chip, microfluidics, fluid automation, micro-bioreactors, smart fluidics

## Abstract

In this work, we developed and characterized a novel fluidic platform that enables long-term in vitro cell culture in a semi-automated fashion. The system is constituted by a control unit provided with a piezoelectric pump, miniaturized valves, and a microfluidic network for management and fine control of reagents’ flow, connected to a disposable polymeric culture unit resembling the traditional multiwell-like design. As a proof of principle, Human Umbilical Vein Endothelial Cells (HUVEC) and Human Mesenchymal Stem Cells (hMSC) were seeded and cultured into the cell culture unit. The proliferation rate of HUVEC and the osteogenic differentiation of hMSC were assessed and compared to standard culture in Petri dishes. The results obtained demonstrated that our approach is suitable to perform semi-automated cell culture protocols, minimizing the contribution of human operators and allowing the standardization and reproducibility of the procedures. We believe that the proposed system constitutes a promising solution for the realization of user-friendly automated control systems that will favor the standardization of cell culture processes for cell factories, drug testing, and biomedical research.

## 1. Introduction

Traditional in-vitro techniques for cell culture allow the growth and proliferation of cells in an artificial and controlled environment and play a key role in elucidating physiology, disease mechanisms, and in testing new drugs [1,2,3,4]. These well-established approaches, which rely on 2D culture of cell lines and primary cells in standard Petri dishes and flasks, are still the most diffused in biology and biotechnology research, as well as in the pharmaceutical industry, cell factories, and other biomedical application areas [5].

Nowadays, cell cultivation practices are not very different from those used on a large scale in the second half of the 20th century [6]. Although these procedures enabled the understanding of a number of phenomena concerning cellular life and activity and proved to be determinants for applications such as drug testing and tissue regeneration, they present several intrinsic limitations. In fact, traditional cell culture systems require numerous and complex manual handling methods, limiting the standardization of the cultivation in terms of cell yield and activity [7]. Moreover, long-term cell maintenance is expensive and time consuming, requiring high quantities of reagents, test samples, and effort. The transition to user-friendly automated devices would favor the standardization of the cultivation process and the reduction of production costs and preparation times for cell culture through the decrease of the number of interventions by the operator [8].

Microfluidics and lab-on-a-chip technologies constitute a strategical solution to favor this transition. Performing cellular assays in microfluidic devices provided the advantages of reduced reagent consumption by replacing traditional Petri dishes and flasks with micro-culture systems, offering a cost-effective route for high-throughput cell production and culture in a controlled environment [9]. The micro-bioreactor is a simple yet archetypal microfluidic device that operates similarly to a standard multi-well plate, but with integrated fluidics circuits [10]. The design of this system is an array of cylindrical culture chambers connected to a network of micro- and/or millimeter-sized channels for the injection and withdrawal of cell media and fluids of interest for a specific cell assay [11]. Soft lithography and polymer micro-fabrication technologies allowed for the rapid prototyping of such devices based on polymeric biocompatible materials, such as polydimethylsiloxane (PDMS), polyethylene glycol diacrylate (PEGDA), hydrogels, and thermoplastics [12,13,14,15]. The in-vitro culture in fluidics micro-bioreactors has been reported in the literature, both in static and dynamic conditions, for a variety of cells, including stem cells, immortalized tumor cells (HeLa), cardiomyocytes and skeletal muscle cells of murine myoblasts, CrFk-type fibroblasts, and HN9.10e neuronal cell models [16,17,18,19].

At the present time, the operation of such micro-devices is restricted to the use of relatively complex, specific, and bulky external equipment and macro-to-micro interface systems for fluidic control, requiring the constant contribution of human operators to manage fluids flow in a manual and not standardized manner [20,21,22]. These approaches recently stimulated a paradigmatic question in the lab-on-a-chip community about the effective impact of microfluidic devices in standard cell culture: are we dealing with “lab-on-a-chip” or “chip-in-a-lab” systems? [23]. The usability and readout reliability of micro-bioreactors would massively benefit from the systematic implementation of a suitable integrated control system for precise fluid handling in a remote control framework. This is a key factor to render cell culture protocols automatic, minimizing the operator’s contribution, and to drastically reduce the number of connection tubes and inlet and outlet ports and external bulky equipment. 

Several works have been reported in the literature concerning automated cell culture on-chip, as automation is widely recognized as a benchmark parameter in modern microfluidic systems for cell biology [24,25,26]. At the research laboratory level, the minimization and miniaturization of additional peripheral equipment is considered a key requirement for the usability of automated devices in cell culture, along with the need for systems that are ready-to-use in a plug-and-play manner [24,27]. Representative examples of microfluidic platforms that target fulfilling these requirements and that are already on the market are the automation systems produced by LabSmith (uProcess™), PreciGenome (iFlow Touch™), and ElveFlow (Liquid Handling Pack). To our knowledge, these and other similar commercial systems only focus on providing the source for a pressure-driven flow. In order to realize a more complex fluidic system to achieve high-integration and high-parallelization (i.e., increasing system compactness and enabling the precise management of multiple channels), there would be a large number of tubes and interconnections involved, as well as relatively large valves, increasing the overall device dimension, and making the platform setup and usage cluttered and error prone. 

Here, we present a novel fluidic platform for semi-automated cell culture into fluidic miniaturized bioreactors, prototyped based on a traditional multiwell-like design. The platform is composed of an electronic system equipped with integrated micro-pumps, valves, and microfluidic circuits for the management and fine control of reagents’ flow and a disposable polymeric cell culture unit provided with fluidic channels and culture sites. This system combines the features of standard cell culture techniques (monolayer culture in cylindrical wells) with the possibility to conduct specific culture protocols remotely, simplifying the entire process, minimizing the contribution of human operators, and allowing the systematic culture of complex cell systems. At odds with what is on the market and reported in the literature, our fluidic system presents active valves interlocked on a fluidic backplane with relatively complex interconnections. The combination between the complexity of the fluidic design and the use of embedded micro-valves actuated by shape memory alloys actuators renders the system compact and highly performing in managing multiple fluidic channels suitable for cell culture protocols. Our fluidic platform was tested on two different primary human cell types, Umbilical Vein Endothelial Cells (HUVEC) and Mesenchymal Stem Cells (hMSC). The cells were cultured in a semi-automated fashion and morphology, known to predict cell health, which was analyzed by confocal microscopy. In addition, the proliferation rate of HUVEC and the osteogenic differentiation of hMSC were investigated. All the data obtained were compared to the cells cultured in standard culture dishes.

### System Design and Semi-Automated Culture Approach

**Figure 1:**
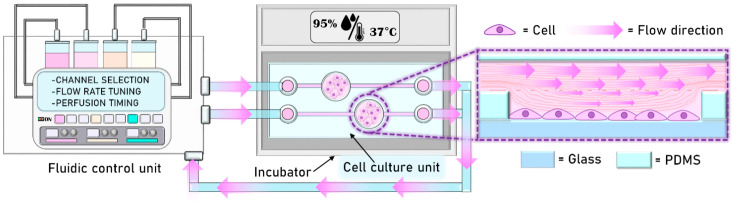
Schematic representation of the microfluidic control platform, including the control module and disposable PDMS unit.

The system (Figure 1) consists of two main units: (i) a fluidic control unit, for the fine control; transport; and perfusion of reagents, culture media, and cells within the system. The fluid transportation takes place within an integrated polymeric fluidic platform, supplied with a network that includes several precision valves and a piezoelectric pump of micro-channels for the handling of the liquids of interest. (ii) A disposable multi-well like cell culture unit, produced in polydimethyilsiloxane (PDMS) via soft-lithography, connected to the fluidic unit through silicone tubes. 

The feasibility of the fluidic platform and PDMS-based device for semi-automated on-chip cell culture was assessed using HUVEC and hMSC as test cellular models. In both cases, the culture protocol operations, such as the change of culture medium and the injection of reagents of interest, were timed and controlled externally via the fluidic control unit, while the fluidic cell culture unit was positioned in an incubator at 37 °C and 5% CO_2_. We chose 500 μL/min as the flow rate, providing a laminar flow and low values of shear stress during the assays. With this setup and conditions, we were able to perform a 3-days-long cell culture experiment for HUVEC and a 4-days-long culture for hMSC.

## 2. Materials and Methods

### 2.1. Fluidic Control Unit

**Figure 2:**
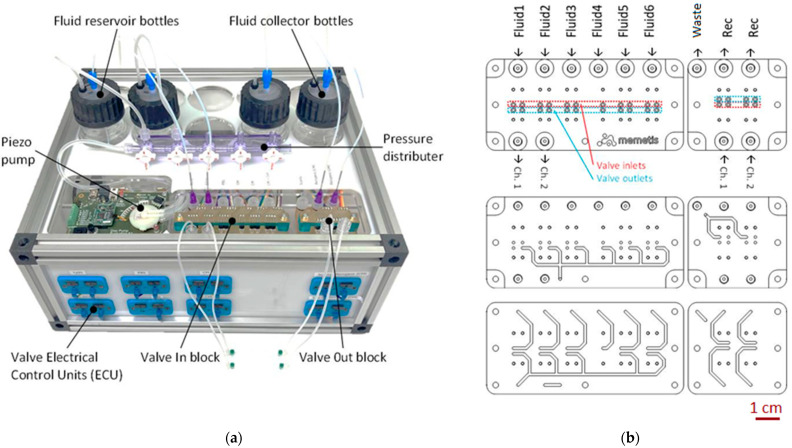
(**a**) Fluidic control unit for cell culture integrated in a housing; (**b**) schematic top view of fluid distribution circuit (top); cut view of first channel level (middle); cut view of second channel level (bottom). The system is designed to distribute different input fluids to the fluidic cell culture unit and from there to collector bottles. The fluids are transported by pressure-driven flow.

Implemented by Dolphin Fluidics in cooperation with memetis GmbH, the fluidic control unit is a pressure-driven flow system for fluid transportation and delivery. The source fluids are stored in six chemically inert glass vessels (fluid reservoir bottles) in the back of the fluidic control unit (Figure 2a). These vessels can be pressurized by a single piezoelectric air pump from TTP Ventus Ltd. (Royston, UK), which operates completely silently. A thin PTFE tube (inner diameter 0.8 mm) also reaches through the cap of each vessel, down to its bottom. This way, the internal air pressure will drive fluid from the vessel through the tube when its external end is open. 

Distribution of the six supply fluids to the inlets of the cell culture platform is managed by a custom 3D-printed biocompatible manifold within the fluidic control unit (Figure 2b), which has two internal levels of flow paths. Eleven normally closed microvalves “Series 09” by Memetis are flange-mounted to the manifold to allow automatic control of fluid pathways (“in-block”). Fluids that leave the fluidic platform are handled by four additional valves (“out-block”) on the same manifold and directed to three collector vessels. The schematic diagram of the fluidic circuit is reported into the Supplementary Material File.

The control electronics for the silently operating miniature valves, integrated in the front part of the unit, support automated valve control via USB connection and I2Cprotocol, as well as manual operation using toggle buttons in the front panel. Due to their functional principle, the valves do not only support fully closed/fully open switching states, but can also adopt intermediate states to enable precise flow rate adjustments via closed-loop control. The maximum pressure achievable with this system is 2 bar, while the maximum flow rate value is 80 mL/min, which can be obtained at a pressure of 1 bar. Both pressure and flow rate resolution are +/−2%.

The extreme compactness of the valves with an installation pitch of only 5 mm is enabled by a fatigue-free shape memory alloy (SMA) thin film actuator, allowing for more than 20 million switching cycles. A low-power consumption of 0.15 W holding power (0.3 W switching power) per valve assures that heat introduction into the controlled media is minimal.

### 2.2. Disposable Cell Culture Unit Fabrication

The disposable cell culture unit was produced by means of soft-lithography, using micro-molding of PDMS, a soft polymer, typically employed for prototyping this kind of fluidic system. In fact, PDMS is an elastomeric polymer with suitable properties for biomedical applications, including physiological indifference, excellent resistance to biodegradation, biocompatibility, chemical stability, gas permeability, excellent optical transparency, and can be easily processed using replica molding [28,29,30]. 

**Figure 3:**
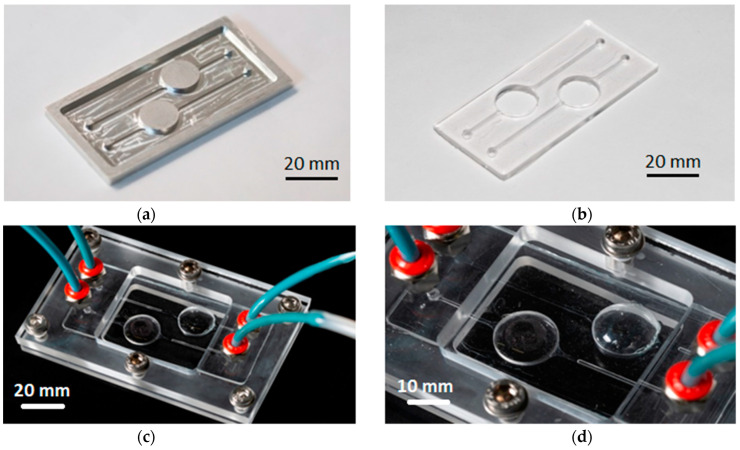
(**a**) Master obtained by micro-milling aluminum components, resembling the final cell culture unit’s microstructures; (**b**) cell culture unit, sealed and bonded to a PDMS membrane (upper part) and to a glass coverslip (bottom part); (**c**) final PDMS cell culture unit sandwiched between PC layers. PC were provided with inlet and outlet ports, positioned to match the ends of the PDMS cell culture unit, machined to accommodate standard fittings for the four ports for flowing fluids through the cell culture unit; (**d**) close-up view of the cell culture chambers, sealed on top with the oxygen-permeable PDMS membrane.

Suitable master molds (Figure 3a) were fabricated by numerically controlled micro-milling of aluminum slabs to obtain protruding microstructures, resembling the final device design (Figure 3b–d). The disposable unit is designed with cylindrical culture wells with a 15 mm diameter, typical of that of a standard 24 multi-well plate, and is provided with micro-fluidic channels with a submillimeter-sized cross section (800 μm) for fluid transportation into the culture sites. The channels and chamber height were dimensioned to enable low flow rates (100 to 500 µL/min) with associated low values of shear stress affecting the cultured cells during chamber perfusion. 

PDMS (Sylgard 184 Silicone Elastomer Kit, Daw Corning) was cast over the master to replicate the fluidic network according to the following protocol. The elastomeric part was mixed with a curing agent at a standard ratio of 10:1 *w*/*w*. The mixture was stirred for 20 min and placed in a vacuum desiccator to remove air bubbles. The PDMS was then poured into the aluminum master and placed in oven for about 2 h and 15 min at 80 °C. After fabrication, the PDMS platform was bonded to 0.1-mm-thick glass coverslips using oxygen plasma treatment to seal the bottom part of the channels. The treatment was performed inside a low-pressure Zepto Plasma Surface treatment machine, purchased from Diener Electronic GmbH (Ebhause, Germany). The oxygen pressure inside the chamber was fixed at 1.0 mbar, and the surface treatment was applied at a power of 80 W for 1 min and 30 s. Surfaces were bonded together by direct contact, applying pressure manually.

A 0.03-mm-thick PDMS membrane serving as the roof for the culture wells was produced by spin coating. This specific thickness allows for oxygen exchange between the cell culture chambers and the incubation environment [31,32]. The PDMS-membrane was stacked over the cell culture unit via the same optimized oxygen plasma treatment (Figure 3b). The PDMS cell culture unit was then finally sandwiched into a manifold, consisting of two fastened polycarbonate (PC) frames produced by means of CNC milling, in order to ensure fluidic seal of the system (Figure 3c).

### 2.3. Semi-Automated Cell Culture

The device has two independent cell culture chambers of 1.9 cm² surface area and containing 500 μL of medium and cells. Cell cultivation took place directly on glass, since no cell adhesion coatings were added at the bottom of the culture chambers. In order to ensure sterilization and removal of circulating bubbles, we preliminarily fluxed 70% ethanol inside the chip, connected to the machine, and in each channel of the fluidic platform. We then exposed the whole system to UV-c bactericidal action for 30 min, inside the biological hood. Each beaker, inside the platform, was preliminarily. We next proceeded to seed cells inside the chip, linking it to the microfluidic control platform through silicone tubes: The microfluidic control platform enabled us to perform semi-automated cells seeding in a controlled manner at a flow rate of 500 μL/min. These procedures are strictly carried out inside the biological hood, except for the sterilization and bubbles removal, which can take place on a lab bench.

#### 2.3.1. Culture of HUVEC

HUVEC were purchased from Lonza (Basel, Switzerland) and cultured at 37 °C and 5% CO_2_ in EBM-2 medium (Lonza), supplemented with 10% fetal bovine serum and EGM-2 SingleQuots Supplements (Lonza). They were used up to passage 4. 10⁴ HUVEC were seeded on each chamber of the cell culture unit and on glass coverslips (13 mm in diameter) allocated on a 24-well plate as control. After 3 days, the cells were trypsinized via microfluidic control platform, in a semi-automated manner, collected in the collector bottle, and counted using a cell counter. The experiment was repeated five times in duplicate. At the end of the experiments, some samples were fixed for confocal analysis.

#### 2.3.2. Culture of hMSC

hMSC were purchased from CliniSciences and were cultured at 37 °C and 5% CO_2_ in Dulbecco’s Modified Eagle’s Medium containing 10% fetal bovine serum and 2 mM glutamine (culture medium, CM). All the reagents for hMSC culture were from Sigma-Aldrich. The cells were used between passage 2 and 6. In total, 3.5 × 10⁴ hMSC were seeded on each chamber of the cell culture unit and on glass coverslips as control. To induce osteogenic differentiation, once the cells were confluent, the CM was changed via microfluidic control platform with an osteogenic medium (OM) containing 2 × 10^−8^ M 1α,25-Dihydroxyvitamin D3, 10 mM β-glycerolphosphate, and 0.05 mM ascorbic acid (Sigma-Aldrich, St. Louis, MO, USA). After 24 h of culture, the cells were trypsinized and collected for Real-Time PCR or fixed for confocal analysis.

#### 2.3.3. Real-Time PCR

Total RNA was extracted by the PureLink RNA Mini kit (Thermo Fisher Scientific, Waltham, MA, USA). Single-stranded cDNA was synthesized from 0.3 μg RNA in a 20 μL final volume using High Capacity cDNA Reverse Transcription Kit, with RNase inhibitor (Thermo Fisher Scientific) according to the manufacturer’s instructions. Real-time PCR was performed on 20 ng of cDNA using TaqMan™ Fast Universal PCR Master Mix (Thermo Fisher Scientific, Waltham, MA, USA) and TaqMan Gene Expression Assays (FAM) (Thermo Fisher Scientific, Waltham, MA, USA). The following primers were used: RUNX2 (Hs00231692_m1), Sp7 (Hs01866874_s1), COL1A1 (Hs00164004_m1), and SPP1 (Hs00959010_m1). The housekeeping gene GAPDH (Hs99999905_m1) was used as an internal reference gene. The reactions were performed with CFX96 Real-Time PCR Detection System (Bio-Rad, Hercules, CA, USA). Relative changes in gene expression were analyzed with the 2^−ΔΔCt^ method.

#### 2.3.4. Confocal Microscopy

HUVEC and hMSC were fixed in phosphate buffered saline containing 4% paraformaldehyde and 2% sucrose pH 7.6 and permeabilized with Triton 0.3%. HUVEC were incubated with anti-cyclophilin (CYP) D and anti-VE-Cadherin overnight at 4 °C, and stained with Alexa Fluor 488 and 647 secondary antibodies respectively (ThermoFisher Scientific, Waltham, MA, USA). hMSC were incubated with anti-CYP D overnight at 4 °C, and stained with Alexa Fluor 488 secondary antibodies (ThermoFisher Scientific, Waltham, MA, USA). Rhodamine-labeled phalloidin was used to visualize the cytoskeleton. 4′,6-Diamidine-2′-phenylindole dihydrochloride (DAPI, Sigma) was used to stain the nuclei. Finally, cells were mounted with ProLong Gold Antifade Mountant (Invitrogen, Carlsbad, CA, USA) and images were acquired using a 40× objective in oil by a SP8 Leica confocal microscope.

#### 2.3.5. Statistical Analysis

Data are expressed as the mean ± standard deviation. The data were analyzed using one-way ANOVA. Statistical significance was defined as *p*-value ≤ 0.05. 

## 3. Results & Discussion

**Figure 4:**
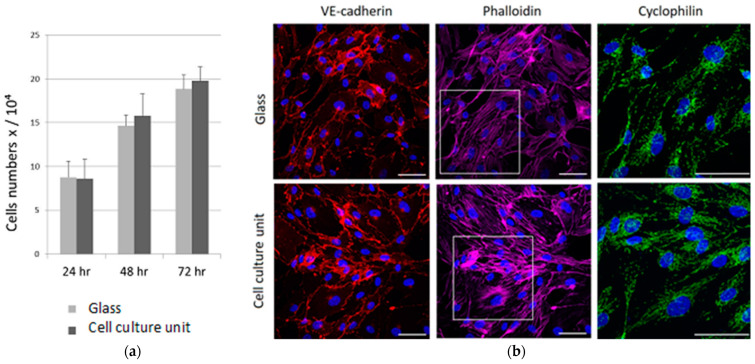
HUVEC were seeded via microfluidic control platform on cell culture unit or on glass coverslips as control. (**a**) The cells were counted using an automated cell counter. Data are the means ± standard deviation of five independent experiments in duplicate. (**b**) HUVEC were cultured on cell culture unit and on glass coverslips for 72 h. The cells were fixed and observed by confocal microscopy after phalloidin, Ve-cadherin, cyclophilin F, and DAPI staining. White square is zoomed-in cyclophilin staining image. Scale bar: 50 µm.

The validation of the disposable cell culture unit and the fluidic control platform was assessed by culturing two different cell types, HUVEC and hMSC. HUVEC were cultured for 3 days and the proliferation rate and imaging analyses were performed. hMSC were cultured for 3 days in CM till they reached the confluence and for an additional day in the presence of the OM to induce the osteogenic differentiation. Osteogenic marker expression and imaging analyses were then performed. Both cell cultures were performed in a semi-automated fashion, providing fresh medium exchange every 24 h in a semi-automated manner at 500 μL/min flow rate. We selected this flow rate value according to the channels geometry and dimensions in order to keep low shear stresses in the culture chamber during perfusion [33,34]. As shown in Figure 4, HUVEC cultured in the cell culture unit grows as fast as the cells cultured on a standard glass coverslip. Taken together, these results suggest that the design of our cell culture unit provides the proper amount of gas and nutrients exchange between cultured cells and inner/outer environments, allowing them to grow and proliferate according to standard culture on wells. Moreover, the use of precision miniaturized valves and a pulsation-free pump embedded in the control unit is crucial for avoiding harsh shear stress during medium perfusion and to keep a laminar flow throughout the performed assays, avoiding fluidic-mechanical stresses on the cell cultures. 

By confocal microscopy, we analyzed HUVEC actin cytoskeleton. Figure 4b demonstrates that the cells cultured on cell culture unit maintain the same polygonal morphology of the control cells seeded on glass coverslips.

In both samples, the actin is organized as a network of well-defined stress fibers spanning the entire cell body. We also analyzed the vascular endothelial (VE)-cadherin, which is an endothelial-specific adhesion molecule located at junctions between endothelial cells. A correct VE-cadherin-mediated adhesion is fundamental not only to control vascular permeability but also to modulate cell proliferation and apoptosis [31]. As shown in Figure 4b, VE-cadherin is well expressed at the cell junctions of both samples. 

Then we focused on mitochondria analysis since their morphology reflects the function of the organelles [35,36] and is continuously modified in response to the different functional requirements of the cell. The anti-CYP D antibody highlights a complex mitochondrial network with elongated mitochondria in HUVEC cultured in the cell culture unit and on a traditional coverslip (Figure 4b).

**Figure 5:**
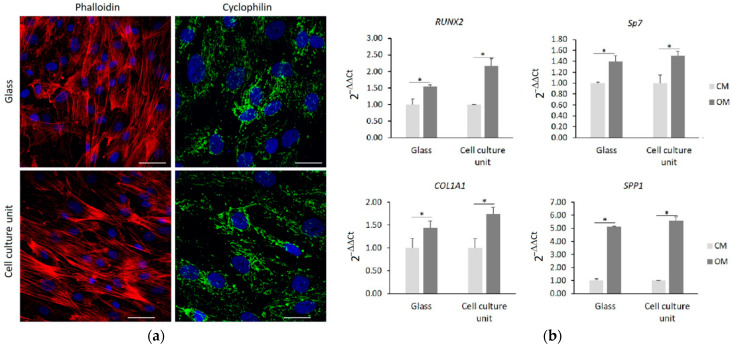
hMSC were cultured on cell culture unit and on glass coverslips. (**a**) After 72 h, cells were observed by confocal microscopy after phalloidin, cyclophilin F, and DAPI staining. Scale bar: 50 µm. (**b**) Confluent hMSC were cultured for 24 h in OM. Real-Time PCR was performed on RNA extracted from hMSC using primers designed on *RUNX2*, *Sp7*, *COL1A1*, and *SPP1* sequence (* *p* ≤ 0.05).

We also tested hMSC as a model to investigate cell differentiation in response to specific stimuli. Initially, the cells were cultured in CM for 3 days in the chip and on a glass coverslip, and then analyzed by confocal microscope. Figure 5a confirms the similar cellular morphology of the two samples with a well-organized actin fiber-network. In both samples, the mitochondria appeared elongated, forming a complex network. 

Once the cells reached the confluence, the medium was changed with the OM and the hMSC were cultured for an additional 24 h. Then, by real time PCR we analyzed the expression of some osteogenic markers. In particular, we analyzed *RUNX2*, which is the first gene to be expressed during osteogenesis, and *Sp7* and collagen 1A1 (*COL1A1*), which are involved in the early stages of differentiation and osteopontin (*SPP1*), which is one of the most abundant non-collagenous components of the bone extracellular matrix. Figure 5b shows that 24 h of culture in the presence of the osteogenic cocktail induces a similar upregulation of all the osteogenic markers both in the cells cultured on the cell culture unit and in the cells cultured on glass coverslips.

According to these results, the culture system proposed proved to be efficient and time saving, reproducing in a semi-automated framework the traditional culture and analysis protocols in a standard multi-well plate-like disposable culture unit. This system will allow to perform different experiments and assays in parallel, scaling-up for high-throughput cell culture processes in a controlled manner. In fact, the control unit offers the possibility to choose a unique and personal set of media/reagents perfusion time, flow rate, and static/dynamic culture conditions. Moreover, the integrated platform avoids the use of bulky and highly specific equipment and interfaces, while reducing to the minimum the complexity of assembling the units. The optimization of this platform, in view of a full automation of the process, will also allow to culture and co-culture complex biological systems (e.g., primary cells, stem cell lines and organoids) without the request for human operators’ contribution. The combination of these features makes this automated culture system a promising solution to implement automation in cultivation processes for cell factories, cells-based precision medicine, and biomedical research.

## 4. Conclusions

In this study, we developed and evaluated the suitability of a novel fluidic platform for the semi-automated culture of cells into miniaturized devices. The platform comprises two main units: a fluidic control unit for the fine control and perfusion of reagents, and a disposable multi-well like cell culture unit produced in PDMS via soft-lithography. HUVEC and hMSC were seeded via the fluidic platform in a semi-automated fashion, setting the proper timing and flow rate, in order to avoid harsh shear stress on cells during media perfusion and to keep a laminar flow throughout the performed assays. In the fluidic system, the cells are vital and maintain their morphology. The HUVEC proliferation rate and the hMSC osteogenic differentiation potential are comparable to the respective control cells cultured on glasses coverslips

The semi-automated approach for cell culture reported here offers several advantages, such as the reduction of time, reagents, and media consumption, and enables the reproduction of traditional culture and analysis protocols using a standard multi-well plate-like disposable culture unit, massively decreasing the operators’ efforts. We plan to develop and optimize a fully automated system to perform remotely different cultures and assays in parallel, scaling-up the system for a high-throughput culture process in a time saving and cost-effective manner. In this way, the entire procedures will be simplified, minimizing the contribution of human operators and allowing the standardization and reproducibility of the in-vitro culture of complex cell systems, such as organoids.

## Supplementary Materials

The following supporting information can be downloaded at: https://www.mdpi.com/article/10.3390/mi13070994/s1, Figure S1: Schematic diagram of the fluidic circuit of the control platform.
