# Supplementary material for: A Novel Fluidic Platform for Semi-Automated Cell Culture into Multiwell-like Bioreactors
Source: Micromachines (Basel). 2022 Jun 24;13(7):994. doi: 10.3390/mi13070994 (PMC9316907; doi:10.3390/mi13070994)
Supplement: Supplementary file 1 [file micromachines-13-00994-s001.zip › micromachines-1774297-supplementary.pdf]

A novel fluidic platform for semi-automated cell culture into multiwell-like bioreactors

Francesca Maria Orecchio[1], Vito Tommaso[2], Tommaso Santaniello[1,*], Sara Castiglioni[2], Federico Pezzotta[1], Andrea Monti[3], Francesco Butera[3], Jeanette Anne Marie Maier[1,2], Paolo Milani[1,]

[1] Interdisciplinary Centre of Excellence for Nanostructured Materials and Interfaces (C.I.Ma.I.Na.), Department of Physics, University of Milan, Via Giovanni Celoria, 16, 20133 Milan.

[2] Department of Biomedical and Clinical Sciences "L. Sacco", University of Milan, Via Giovanni Battista Grassi 74, 20157 Milan.

[3] Dolphin Fluidics S.r.l., Via Leonardo Da Vinci, 40, 20094 Corsico (MI).

* Correspondence: TS Tommaso.santaniello@unimi.it; Tel.+39-2-50317437;

Supplementary Material

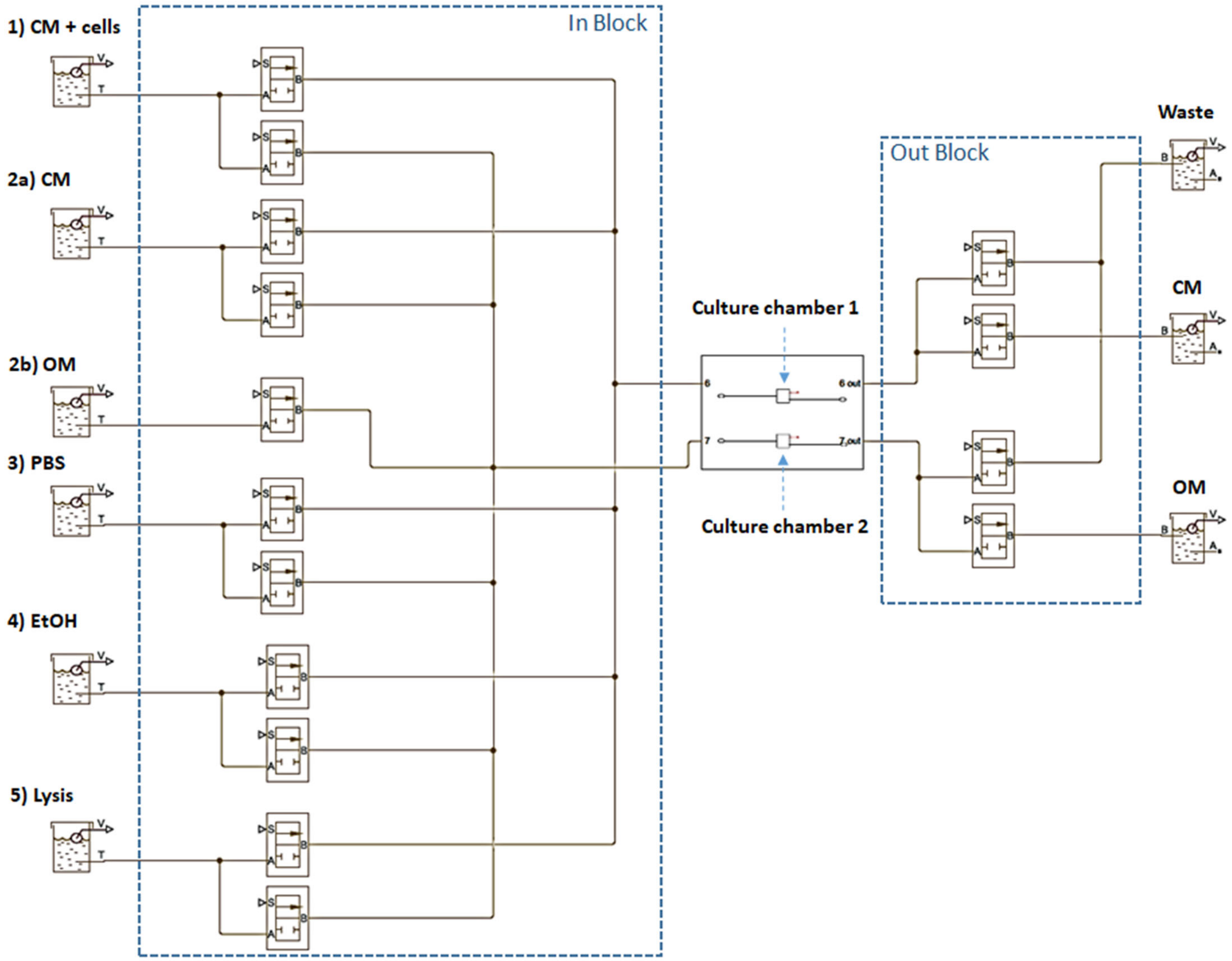

Figure S1. Schematic diagram of the fluidic circuit of the control platform.
